# Selecting patients for randomized trials: a systematic approach based on risk group

**DOI:** 10.1186/1745-6215-7-30

**Published:** 2006-10-05

**Authors:** Andrew J Vickers, Barry S Kramer, Stuart G Baker

**Affiliations:** 1 Department of Epidemiology and Biostatistics, Memorial Sloan-Kettering Cancer Center, New York, USA; 2 Office of Disease Prevention, National Institutes of Health, Bethesda, Maryland, USA; 3 Biometry Research Group, Division of Cancer Prevention, National Cancer Institute, National Institutes of Health, Bethesda, Maryland, USA

## Abstract

**Background:**

A key aspect of randomized trial design is the choice of risk group. Some trials include patients from the entire at-risk population, others accrue only patients deemed to be at increased risk. We present a simple statistical approach for choosing between these approaches. The method is easily adapted to determine which of several competing definitions of high risk is optimal.

**Method:**

We treat eligibility criteria for a trial, such as a smoking history, as a prediction rule associated with a certain sensitivity (the number of patients who have the event and who are classified as high risk divided by the total number patients who have an event) and specificity (the number of patients who do not have an event and who do not meet criteria for high risk divided by the total number of patients who do not have an event). We then derive simple formulae to determine the proportion of patients receiving intervention, and the proportion who experience an event, where either all patients or only those at high risk are treated. We assume that the relative risk associated with intervention is the same over all choices of risk group. The proportion of events and interventions are combined using a net benefit approach and net benefit compared between strategies.

**Results:**

We applied our method to design a trial of adjuvant therapy after prostatectomy. We were able to demonstrate that treating a high risk group was superior to treating all patients; choose the optimal definition of high risk; test the robustness of our results by sensitivity analysis. Our results had a ready clinical interpretation that could immediately aid trial design.

**Conclusion:**

The choice of risk group in randomized trials is usually based on rather informal methods. Our simple method demonstrates that this decision can be informed by simple statistical analyses.

## Background

Protocols of randomized trials specify inclusion and exclusion criteria to determine the population under study. Exclusion criteria typically focus on identifying subjects who might be harmed by the study intervention, those for whom benefit is doubtful and those who are unlikely to provide useful data. Inclusion criteria tend to focus on risk: all trials identify the population at risk for the study event, some trials additionally specify criteria to define a study population at high-risk. For example, a trial comparing recurrence rates between two approaches to prostatectomy will specify only that patients with localized prostate cancer are eligible; a trial to determine the effects of adjuvant therapy might further restrict eligibility to patients with locally advanced cancer who are at high risk of recurrence. In some cases trialists studying a similar question have reached different conclusions as to whether to include the whole at-risk population or only a high-risk subgroup. The PLCO trial, for instance, includes all older individuals in a study of lung cancer screening [1], whereas the National Lung Screening Trial[2] includes only smokers, or recent quitters, with a smoking history of 30 pack-years or more.

In this paper, we present a simple statistical approach for determining whether trialists should use a "whole population" or "high-risk group" approach. The results of the method have a ready clinical interpretation that can immediately aid trial design, moreover, the method is easily adapted to determine which of several competing definitions of high-risk is optimal. In a previous paper, which focused on screening and prevention, two of us argued that the population accrued to a trial should be the same as that to whom the intervention will be applied in practice[3]. Thus the decision whether to include all members of an eligible population, or just a high-risk sub-group, should depend on the relative benefit of these alternative strategies were they to be clinically applied after the completion of a trial indicating an effect of the intervention. We therefore model benefits of alternative strategies in a hypothetical population in order to determine the optimal approach.

## Method

Our method is based on the assumption that interventions proven in randomized trials will be offered to eligible patients similar to those studied in the trial. For example, we assume that if a trial accruing patients with locally advanced prostate cancer demonstrates effectiveness of adjuvant therapy, such treatment will subsequently be offered to patients with locally advanced but not organ-confined disease. We therefore compare the predicted outcome of treating all at-risk patients in the population at large to the outcome of treating only the high-risk subgroup. We then recommend the approach with the better outcome to determine the inclusion criteria for the randomized trial.

Outcome is defined in terms of "net benefit" in the eligible population. Net benefit is a concept often used in economic analysis and is simply benefits minus harms. In the case of a medical intervention, "benefits" are associated with reduction in the event rate compared to no additional treatment: in an adjuvant therapy trial, for instance, benefit would be a reduction in cancer recurrences or deaths compared to surgery alone. "Harms" are associated with the intervention itself: side-effects, costs, inconvenience and so on. To assess the relative outcome of the whole population and high-risk approach, we therefore need to calculate the proportion of patients who would be treated, and the reduction in event rate, for each approach. For the whole population approach this is straightforward: the proportion of patients treated is 100% and the reduction in event rate is simply the event rate in the absence of intervention multiplied by the anticipated relative risk of the event with versus without intervention.

To determine intervention and event rates for the approach of treating only high-risk patients, consider that an investigator proposing a trial on high-risk patients must propose specific criteria to determine high-risk. This might involve a single risk factor (e.g. non-organ confined disease) or a threshold probability on a multivariable prediction model (or "nomogram") including a variety of risk factors (e.g. stage, grade, PSA [4]). Such criteria can be seen as a prediction tool: individuals who meet the criteria are thought to be more likely to experience a disease-related event than those who do not meet the criteria. We can then calculate the sensitivity and specificity of this prediction tool. To define "sensitivity" we use as the numerator the number of individuals in the eligible population who both have the event (in the absence of intervention) and who are classified as high risk; the denominator is the total number of patients who have the event. "Specificity" is comparably defined by using for the numerator the number of individuals in the eligible population who do not have the event and who do not meet the criteria for high risk; the denominator is the total number who do not have the event (see table 1 for illustrative data). Data on sensitivity, specificity and event rate may be obtained from epidemiologic data sets or from analyzing the control arm of prior randomized trials.

**Table 1:** Relationship between criteria for defining a "high-risk" sub-group and whether a patient has an event during a clinical trial.

		**Event?**	
		
		**Yes**	**No**
**Meet criteria for "high-risk"?**	**Yes**	396	624
	**No**	632	3,799

Hypothetical data are given for the purposes of illustration.

Sensitivity = 396 ÷ (396+632) = 39%

Specificity = 3799 ÷ (624+3799) = 86%

In the Appendix [see Additional file 1], we derive the following formulae for the intervention and event rates when selecting a high-risk group.

**Intervention rate: **the anticipated proportion of eligible population receiving intervention when the intervention is given only to high risk subjects

= Event rate in the absence of intervention × sensitivity + (1 – Event rate in the absence of intervention) × (1 – specificity)

**Event rate: **the anticipated proportion of eligible population who have the event when intervention given to the high risk group:

= Event rate in the absence of intervention × sensitivity × relative risk + Event rate in the absence of intervention × (1 – sensitivity)

Decrease in event rate due to intervention:

= Event rate in the absence of intervention – Event rate when intervention given to high risk group

Where:

**Event: **a negative medical outcome such as disease recurrence or death occurring within the projected time frame of the trial

**Event rate in the absence of intervention: **expected proportion of individuals in the eligible population who will have the event

**Sensitivity: **numerator: number of individuals in the eligible population who both have the event (in the absence of intervention) and who are classified as high risk; denominator: the total number of patients who have the event.

**Specificity: **numerator: number of individuals in the eligible population who do not have the event and who do not meet the criteria for high risk; denominator: total number who do not have the event.

**Relative risk: **relative risk of the event with versus without intervention

As described above, net benefit is benefit minus harm, where benefit is related to the number of events and harm to the number of interventions. To formulate net benefit precisely, it is necessary to put benefits and harms on the same scale. The problem is that events and interventions are not equivalent: an event, such as a prostate cancer recurrence, is generally considered worse than an intervention, such as adjuvant therapy. Just how much worse an event is considered than an intervention will vary from case to case. A common way of converting between events and interventionsis the "number-needed-to-treat" (NNT). We define the threshold NNT (NNT_t_) as the maximum number of patients that a clinician would treat to prevent one event. The NNT_t _may be based on an informal subjective judgment; alternatively, methods have been described in the literature to derive NNT_t _based on the relative harm associated with intervention and an event[5]. NNT_t _can be thought of in economic terms as the amount we would pay, in interventions, to avoid one event. As such, NNT_t _is independent of the event rate. Hence we define:

Net benefit = decrease in event rate – intervention rate ÷ NNT_t_

Note that the units of the left and right terms in the net benefit equation are the same: NNT_t _is in units of intervention rate divided by event rate, so the units are in terms of event rates.

We propose calculating net benefit for the strategy of treating all patients and for treating only the high-risk group. The approach with the highest net benefit in the eligible population after completion of the trial is chosen for trial design.

### Illustrative example

A group of investigators wish to investigate whether adjuvant therapy can reduce the risk of recurrence after radical prostatectomy. They plan to randomize patients to surgery alone or surgery with hormonal therapy and follow patients for five years to determine the proportion who recur. About 20% of all prostatectomy patients recur within 5 years (i.e. the event rate in the absence of intervention) and the expected effect of adjuvant therapy is a relative risk of 0.75. Discussion with clinicians and patients suggest an NNT_t _of 100 for prostate cancer death, that is, if 100 or fewer patients had to be treated with adjuvant therapy to prevent one death, it would be considered worth taking; if more than 100 had to take the agent to prevent one death, the costs, side-effects, risks and inconvenience of the drug would be seen to outweigh its benefits. As only approximately one in three patients who recur after prostatectomy die from disease, the NNT_t _for the study endpoint of recurrence is 33.

The standard predictive model for prostate cancer recurrence is the "Kattan nomogram" and this has been used in several randomized trials to determine eligibility. Trials have varied as to the threshold risk of recurrence used to determine eligibility: 40% for NCT00283062, 50% for NCT00132301 and 25% for NCT00258765. Let us imagine that our group of investigators disagree as to the optimal threshold: whilst one investigator wishes to define patients as "high risk" if they have 50% or greater risk of recurrence, another argues that the threshold should be set much lower, at 10%, in order to ensure that most patients who actually do recur would be eligible. Meanwhile, the drug company argues that prostate cancer is an unpredictable disease and that the investigators should keep an open mind about whether to accrue all prostatectomy patients to the trial. Note that although the Kattan nomogram is a multivariate model, this is not a requirement of our approach: eligibility criteria can be determined by a model, by a single risk factor – such as a smoking history of at least 30 pack-years – or a combination of risk factors, such as including patients with either high stage cancer or a positive surgical margin.

We obtained from the author data on the sensitivity and specificity of the Kattan nomogram at various cut-points. Table 2 gives the intervention rate, event rate, decrease in event rate from intervention and the net benefit for both the different high-risk categories and the strategy of treating all patients. Note that the event rate is not that observed in the trial, but that in the population as a whole, were the intervention to be applied in practice.

**Table 2:** Calculations to determine whether to treat the whole population or just a high-risk group.

**Strategy**	**Sensitivity**	**Specificity**	**Intervention rate**	**Event rate**	**Decrease in event rate (benefit)**	**Net benefit (benefit – intervention rate ÷ NNT**_t_**)**
Treat none	0%	100%	0.0%	20.00%	0%	0
Treat high-risk (risk 10% +)	91%	57%	52.60%	15.45%	4.55%	0.02956
Treat high-risk (risk 50% +)	47%	96%	12.60%	17.65%	2.35%	0.01968
Treat all	100%	0%	100.0%	15.00%	5.00%	0.01970

The relative risk of the intervention is 0.75, the NNT_t _is 33 and the event rate in the absence of intervention is 20%. The decrease in event rate is the event rate in the population in the absence of intervention minus the event rate in the population when individuals meeting the high-risk definition receive intervention.

As a worked example, we will look at the strategy of treating only patients with a risk of 50% or more. The formula for the intervention rate is: Event rate in the absence of intervention × sensitivity + (1 – Event rate in the absence of intervention) × (1 – specificity), i.e., 20% × 47% + 80% × 4% = 12.6%. The formula for the event rate after the intervention is applied to high-risk subjects is: Event rate in the absence of intervention × sensitivity × relative risk + Event rate in the absence of intervention × (1 – sensitivity) or 20% × 47% × 0.75 + 20% × 53% = 17.65%. This is a decrease is event rate of 20% – 17.65% = 2.35%. The formula for net benefit is decrease in event rate – intervention rate ÷ NNT_t _giving 2.35% – 12.60% ÷ 33 = 0.01968 as the net benefit for the strategy of treating only men with a risk of 50% or more.

From the table, we can see that the highest net benefit is associated with treating only men with a nomogram predicted risk of recurrence of 10% or more. We would recommend using this as the eligibility criteria for the trial. One particular advantage of our approach is that net benefit has a simple clinical interpretation in terms of either a decrease in event rate while keeping the intervention rate constant or a decrease in the intervention rate while keeping the event rate constant. For example, the net benefit for the high-risk group is 0.0296 greater than that of not using adjuvant therapy in any patient. Thus the strategy of calculating a prediction for all patients and administering an intervention to those with a predicted risk of recurrence ≥ 10% gives the same net benefit as a strategy (say, a change in surgical technique) that leads to the equivalent of about 3 fewer recurrences per 100 patients without any patients receiving adjuvant therapy. A similar calculation can be conducted to determine the decrease in intervention rate for a constant event rate: in this case, the difference in net benefit is multiplied by the NNT_t_.

### Sensitivity analysis

Any of the inputs required to calculate net benefit can be varied to determine whether this affects which strategy is deemed optimal. The event rate in the absence of the study intervention can usually be estimated (e.g. from cohort studies), and whether it is worth varying sensitivity and specificity will depend on the size and quality of the studies used to estimate these parameters. Hence the two most important sensitivity analyses concern NNT_t _– on the grounds that this is a judgment that can reasonably vary from individual to individual and place to place – and relative risk, on the grounds that this is unknown during trial planning.

**Figure 1:**
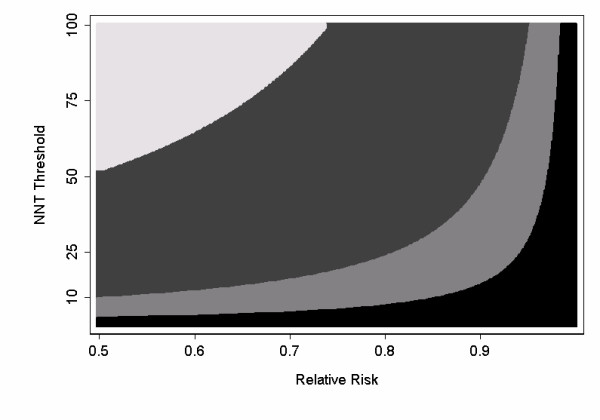
Sensitivity analysis for a prostate cancer adjuvant trial. The shaded areas identify the optimal strategy for each combination of NNT_t _and relative risk. White: include whole at-risk population of men undergoing prostatectomy. Dark grey: Include men with a predicted probability of recurrence ≥ 10% ; Light grey: Include men with a predicted probability of recurrence ≥ 50%; Black: Include no men on the trial (intervention does more harm than good). Note that specificity of the optimal strategy increases from top left to bottom right.

Figure 1 shows the optimal strategy for different combinations of NNT_t _and relative risk. In accordance with intuition, figure 1 shows that the more effective and tolerable the intervention, the more likely we are to selecting intervening in all patients rather than just a high-risk group; the less effective and tolerable the intervention, the more likely we are to chose to treat only a high-risk group, or no-one at all. Let us imagine that one investigator is of the opinion that interventions are rarely as effective or tolerable as hoped. If we reduce relative risk or NNT_t _from the base scenario of 0.75 and 33, we sometimes choose a cut-off of 10% and other times a cut-off of 50%. The investigator therefore suggests examining a cut-off of 25% to define high-risk. This is associated with a sensitivity of 72% and a specificity of 84%. The net benefit for this definition of high-risk is shown in table 3 for various combinations of NNT_t _and relative risk. The new definition is superior for most scenarios. The investigators decide to run the trial using a predicted risk of recurrence of at least 25% as the inclusion criterion for the trial.

**Table 3:** Sensitivity analysis. Net benefit when relative risk and NNT_t _are varied.

		**Relative Risk**			
**NNT**_t_	**Cut-off for risk of recurrence**	**0.75**	**0.8**	**0.85**	**0.9**

10	10%	-0.00710	-0.0162	-0.02530	-0.03440
	25%	0.00880	0.00160	-0.00560	-0.01280
	50%	0.01090	0.00620	0.00150	-0.00320
15	10%	0.01043	0.00133	-0.00777	-0.01687
	25%	0.01787	0.01067	0.00347	-0.00373
	50%	0.01510	0.01040	0.00570	0.00100
20	10%	0.01920	0.01010	0.00100	-0.00810
	25%	0.02240	0.01520	0.00800	0.00080
	50%	0.01720	0.01250	0.00780	0.00310
25	10%	0.02446	0.01536	0.00626	-0.00284
	25%	0.02512	0.01792	0.01072	0.00352
	50%	0.01846	0.01376	0.00906	0.00436
33	10%	0.02956	0.02046	0.01136	0.00226
	25%	0.02776	0.02056	0.01336	0.00616
	50%	0.01968	0.01498	0.01028	0.00558

The relative risk of the intervention is 0.75, the NNT_t _is 33 and the event rate in the absence of intervention is 20%. The decrease in event rate is the event rate in the population in the absence of intervention minus the event rate in the population when individuals meeting the high-risk definition receive intervention.

Our method assumes that, following a positive trial result, all or nearly all high risk patients will receive the intervention, and none, or nearly none, of the low risk population will be treated. This might be seen as a somewhat unrealistic ideal of evidence-based medical practice. However, it is easy to adjust estimates of event rates and intervention rates in the presence of variation from this standard by specifying a proportion of high risk patients are not treated and a proportion of low risk patients who inappropriately receive intervention (see Appendix [Additional file 1] for formulae).

### Applying the method to other sample scenarios

In tables 4 and 5, we create a number of different scenarios to illustrate the circumstances in which it is preferable to select a high-risk group for intervention. Table 4 shows that the value of selecting a high-risk group, in comparison to the whole population approach, is greater as the event rate decreases. In table 5, the value of selecting high-risk patients is associated with lower tolerability or lesser effectiveness of the intervention. If an intervention is either very effective or highly tolerable, the high-risk approach is only of benefit if selection criteria are highly sensitive, in other words, in the case that nearly all those who could benefit from the intervention receive it. Conversely, if the effectiveness of the intervention is moderate, or it is poorly tolerated, selection criteria must be specific, that is, only those patients who would benefit are selected. These considerations suggest that focusing on a high-risk group might be of particular value for screening or prevention trials, as these typically involve low event rates, interventions of moderate effectiveness and a population with a low tolerance for adverse treatment effects.

**Table 4:** Net benefit for treating high-risk and all patients, varying the event rate in the absence of intervention.

**Event rate in the absence of intervention**	**Net benefit (high-risk)**	**Net benefit (treat all)**	**Net benefit compared to treat all**
50%	0.09713	0.12000	-0.02288
10%	0.01803	0.02000	-0.00198
7.5%	0.01308	0.01375	-0.00067
5%	0.00814	0.00750	0.00064
2.5%	0.00319	0.00125	0.00194
1%	0.00023	-0.00250	0.00273

Results are for a scenario where relative risk = 0.75; sensitivity = 80%; specificity = 65% and NNT_t _= 200

**Table 5:** Net benefit for treating high-risk and all patients, varying the effectiveness and tolerability of intervention.

**Scenario**	**Relative Risk**	**NNT**_t_	**Sensitivity**	**Specificity**	**Net benefit (high-risk)**	**Net benefit (treat all)**	**Net benefit: high-risk – treat all**
Effective intervention	0.50	100	40%	80%	0.00790	0.01500	-0.00710
Effective intervention, high sensitivity	0.50	100	95%	45%	0.01805	0.01500	0.00305
Highly tolerable intervention	0.75	500	40%	80%	0.00458	0.01050	-0.00592
Highly tolerable intervention, high sensitivity	0.75	500	95%	40%	0.01064	0.01050	0.00014
Adverse intervention	0.75	40	40%	80%	-0.00025	-0.01250	-
Adverse intervention, high specificity	0.75	40	30%	90%	0.00100	-0.01250	0.01350
The ideal intervention, high sensitivity and specificity	0.25	500	95%	90%	0.03534	0.03550	-0.00016
Questionable intervention, poor sensitivity and specificity	0.80	100	51%	51%	0.00019	0.00000	0.00019

The event rate in the absence of intervention is 5% for all scenarios.

The final two rows of table 5 demonstrate the value of a decision analytic approach to the problem of risk group selection. In one scenario, selection criteria that have near perfect sensitivity and specificity are useless because the intervention is highly effective and tolerable, and therefore there is little downside to treating all patients. In another scenario, selection criteria that are only marginally better than random guessing should be used to select a high-risk group because intervening is of extremely marginal benefit.

### Sample size considerations

We can derive additional simple formulae to help determine sample size (see Appendix [Additional file 1]). The proportion of events in the control arm of the trial is: sensitivity × event rate in the absence of intervention ÷ intervention rate. This number can be entered into a standard sample size calculation for a difference between proportions. We can then calculate the number of patients that need to be screened as number of patients in trial ÷ intervention rate. Table 6 gives number of patients in a trial and number to be screened where sample size is calculated assuming a 25% risk reduction from intervention. Sample size varies considerably between samples, although the number of patients who would need to be screened is reasonably constant. As a worked example, we will look at the first row, the strategy of including all patients with a risk of recurrence of 50% or more. The calculation for the intervention rate has already been described above. The formula for the event rate in the control group of the trial is: sensitivity × event rate in the absence of intervention ÷ intervention rate, i.e. 47% (from table 2) × 20% ÷ 12.6% = 74.6%. To calculate the event rate in the treatment arm, this is multiplied by the relative risk, i.e. 74.6% × 0.75 = 55.95%. Using the *sampsi *function on Stata 9.2 (Stata Corp., College Station, Texas), a trial with 90% power to detect a difference at a significance level of 5% between an event rate of 74.6% and 55.95% requires 292 patients. As we include 12.6% of patients on trial (the intervention rate) to obtain 292 patients we would have to screen 292 ÷ 12.6% = 2317.

**Table 6:** Sample size requirements for different scenarios. Sample size is calculated using 90% power and 5% alpha

**Strategy**	**Intervention rate**	**Event rate in control arm of trial**	**Sample size (pts. screened) for relative risk of 0.75**
Treat high-risk (risk 10% +)	52.60%	34.6%	1228 (2335)
Treat high-risk (risk 25% +)	27.20%	52.9%	624 (2294)
Treat high-risk (risk 50% +)	12.60%	74.6%	292 (2317)
Treat all	100.00%	20.0%	2504 (2504)

The general approach we suggest is only based on net benefit in the eligible population after completion of the trial, and does not take into account the sample size considerations. If there is an upper bound on the sample size due to budget constraints, the risk group selected should be that group with highest net benefit among those under consideration that satisfy the budget for the trial. Alternatively, one could consider a more complex calculation of net benefit subject to a constraint on total trial costs[6].

## Discussion

Determining who should receive an intervention is a key aspect of medical practice. It is inevitable that, although some interventions should be applied to all members of an at-risk population (e.g. antibiotics before surgery), others should be restricted to those at high-risk (e.g. β-blockers before surgery). To our knowledge, no previous investigators have described a simple strategy for determining whether a trial should accrue patients selected from the whole at-risk population or only to those from a high-risk subgroup. Moreover, criteria for determining the appropriate definition of high-risk subgroup have not been developed systematically, for example, by quantitatively comparing different definitions.

Accordingly, prior approaches to issues of patient selection in randomized trials have been rather informal. For example, at a National Cancer Institute workshop on risk prediction models, it was reported that participants "repeatedly discussed the use of cancer risk prediction models for high-risk versus population approaches to cancer prevention". Yet the only guidance given was that a population prevention strategy would be optimal unless a predictive model had "high discriminatory power" to identify those who will develop a disease[7]. This begs the question of just how good a model has to be, and omits what we have demonstrated to be the key variables of the underlying event rate, intervention effectiveness and tolerability.

A debate concerning the inclusion criteria for the National Lung Screening Trial is similarly illustrative. In the original trial protocol, the investigators set inclusion criteria of a 30 pack-year or greater smoking history and no more than 15 years since quitting[2]. No clear rationale was given for this threshold. Subsequently, a separate group of investigators created a risk prediction model for lung cancer and argued that, because its predictive properties were well understood, this might be used to select patients for a clinical trial[2]. However, these investigators did not demonstrate clearly that any specific set of criteria derived from their model was superior to those used in the trial.

Given the apparent advantages of our method, we should discuss some of its limitations. One important assumption of the method is that the relative risk for intervention versus no intervention is constant over the choice of risk groups. Generally speaking, we do not know whether this is true: indeed, two of us have previously used possible inconstancy of relative risk to argue against accruing only high-risk patients and then applying the results to the whole eligible population[3]. However, we think there is an important difference between using a certain assumption to help design a trial and using it to make a clinical decision. In case of a clinical decision about treatment, patients could be harmed if assumptions about relative risk do not hold. We would therefore like to avoid any such assumptions. In the case of trial design, any design we choose necessarily involves assumptions, explicit or otherwise, about the relationship between relative and absolute risk. Moreover, these assumptions can be tested once the trial is completed and further research recommended if appropriate.

An apparent disadvantage of our method is that it involves a subjective judgment of NNT_t_, and a prediction as to relative risk. However, these would be needed for other design decisions even if an investigator chose not to follow our recommendations. For example, the NNT_t _is equivalent to the "minimum clinically significant difference" that is used in standard sample size calculations; predictions as to event rates are similarly part-and-parcel of sample size estimation.

An alternative to the approach suggested here would be to conduct a trial including all patients, use the trial data to build a predictive model and then select a high-risk group accordingly. The principal advantage is that we can model treatment benefit, rather than baseline risk, and therefore do not need to make assumptions about a constant relative risk. This approach has been pioneered successfully with respect to adjuvant chemotherapy[8]. However, in practice, clinicians and statisticians are uncomfortable recommending interventions to sub-groups of patients unless these have demonstrated clinical and statistical significance in the primary analysis of a randomized trial. It is quite plausible that an intervention with a modest effect size, or one targeting a moderately prevalent disease, will not show sufficient overall effectiveness in definitive trial and will be dropped from consideration, even though it would be of important benefit to a sub-group of high-risk patients. An illustrative recent example is the Women's Health Initiative study of calcium and vitamin D for fracture prevention. Overall, this study found rather small benefits of supplementation[9], the key conclusion being that treatment "did not significantly reduce hip fracture". Many of the women in this study were at very low risk: for example, 37% of the participants were aged 50 to 59 and the rate of fracture in this sub-group was only about 0.3%. It is entirely possible that supplementation is of important benefit for older women at higher risk of fracture, but that the use of supplementation in the community will decline given the rather negative overall study results.

In this paper, we have introduced a statistical method to determine whether or not to restrict a study to a high-risk population and, if so, to determine which of several competing definitions of high-risk is optimal. Our method is simple and produces results with direct clinical applicability. It should appeal to clinicians since the quantitative results are in concert with clinical intuition. However, we feel that the mathematical details of our method are perhaps less important than our overall message, which includes four main points. First, it may be more rational to focus on high-risk groups than to treat everyone at risk. Second, whether or not to restrict an intervention to a high-risk group is a question that can be informed by data and statistical analyses. It is our impression that current decisions about whom to include in trials have not been statistically based, rather, they appear to have depended on informal judgment. Third, trial eligibility criteria in trials that attempt to identify high-risk subjects (such as pack years in a lung cancer screening trial) can be seen as predictions with certain statistical properties. We have chosen to describe these in terms of sensitivity and specificity, on the grounds that these terms are readily understood by most clinicians. Fourth, we can compare different approaches to trial eligibility using formal statistical analysis. We believe that a more systematic approach to patient selection will maximize the benefits of randomized trials for human health.

## Supplementary Material

Additional file 1 Appendix. The appendix gives the statistical derivation of the formulae used in the text. Click here for file
