# Supplementary material for: Selecting patients for randomized trials: a systematic approach based on risk group
Source: Trials. 2006 Oct 5;7:30. doi: 10.1186/1745-6215-7-30 (PMC1609186; doi:10.1186/1745-6215-7-30)
Supplement: Additional file 1 — Appendix. The appendix gives the statistical derivation of the formulae used in the text. [file 1745-6215-7-30-S1.doc]

**APPENDIX**

Let E denote an event in the absence of intervention.

Let E* denote an event in a patient who received intervention.

Let RG denote risk group.

We define:

P(E) = Event rate in the eligible population in the absence of intervention

Sensitivity= P(RG | E)

Specificity = P(not RG | not E)

As all patients in the risk group receive intervention:

Intervention rate = P(RG)

**Intervention rate**

It is a mathematical identity that

P(RG) = P(RG, E) + P(RG, not E)

= P(RG | E) ×P(E) + P(RG | not E) × P(not E).

By definition:

Intervention rate = Event rate in the absence of intervention × sensitivity +

(1 – Event rate in the absence of intervention) × (1 – specificity)

**Event rate**

Because an event after intervention would have occurred in the absence of intervention,

P(E | E*) = 1. Therefore we can write

P(E*)= P(E| E*) × P(E*) = P(E*, E). (1)

It is a mathematical identity that the right side of (1) is

P(E*, E) = P(RG, E*, E) + P(not RG, E*, E). (2)

Looking at the second term in (2), it is a mathematical identity that

P(not RG, E* , E) = P(not RG, E* | E) × P(E). (3)

Because intervention does not affect the group not at risk, we can write

P(not RG, E* | E ) = P(not RG, E | E) = P(not RG | E)

Thus (3) is

P(not RG, E* , E) = P(not RG | E) × P(E). (4)

Turning to the first term in (2), it is a mathematical identity that

P(RG, E*, E) = P(RG, E* | E) × P(E) = K × P(RG, E| E ) = K × P(RG| E) × P(E), (5)

where

K= P(RG, E* | E) / P(RG, E| E) by definition

              = P(RG, E* | E) / P(RG| E)  by mathematical identity

              = P(E* | RG, E)  by mathematical identity.
            
              = P(E*, E | RG) / P(E | RG) by mathematical identity
                             
              = P(E | E *, RG) P (E *| RG) / P(E | RG) by mathematical identity
             
              = P(E* |RG) / P(E | RG)  since P(E | E*, RG) =1

Thus K is the relative risk for the intervention in subjects in the risk group, i.e., the ratio of the risk of the event among risk-group subjects in the intervention arm to the risk of the event among risk-group subjects in the control arm

Substituting (4) and (5) into (1) and (2) gives

P(E*) = K × P(RG| E) × P(E) + P(not RG| E) ×P(E),

By definition:

Event Rate = Event rate in the absence of intervention × sensitivity × relative risk +

Event rate in the absence of intervention × (1 – sensitivity)

A key assumption here is that K (relative risk) is the same regardless of the criterion for creating the risk group.

**Event rate and intervention rate given practice variation**

Assume that the intervention is given to proportion *p* of high risk patients and *q* of low risk patients. If clinical practice is perfectly evidence-based, *p* =1 and *q* = 0. In the presence of practice variation, 1 > *p* > *q* > 0. In this case, we use:

Intervention rate =

Event rate in the absence of intervention × sensitivity × *p* +

(1 – Event rate in the absence of intervention) × (1 – specificity) × *p* +

Event rate in the absence of intervention × (1 – sensitivity) × *q* +

(1 – Event rate in the absence of intervention) × (specificity) × *q*

Event rate =

Event rate in the absence of intervention × sensitivity × relative risk × *p*  +

Event rate in the absence of intervention × (1 – sensitivity) × (1 – *q*) +

Event rate in the absence of intervention × sensitivity × (1 – *p*) +

Event rate in the absence of intervention × (1 – sensitivity) × relative risk × *q*

**Sample size calculation**

We need to calculate P(E | RG), the probability of an event in untreated patients accrued to the trial.

Using Bayes' rule:

P(E | RG) = P(RG | E) × P(E) / P(RG)

From our definitions:

Event rate in the control group of the trial =

Sensitivity × Event rate in the absence of intervention / Intervention rate
